# Perioperative Pregabalin for Recurrent Intractable Hiccups: A Case Report and Literature Review

**DOI:** 10.1155/cria/6329954

**Published:** 2026-07-11

**Authors:** David Lei, Craig T. L. Yamaguchi, Alastair E. Moody, Jeffrey D. Swenson

**Affiliations:** ^1^ Western University of Health Sciences College of Osteopathic Medicine of the Pacific, Pomona, California, USA; ^2^ Department of Anesthesiology and Perioperative Medicine, Oregon Health and Science University, Portland, Oregon, USA, ohsu.edu; ^3^ Department of Anesthesiology, Perioperative, and Pain Medicine, University of Utah School of Medicine, Salt Lake City, Utah, USA, utah.edu

**Keywords:** hiccup treatment, intractable hiccups, persistent hiccups, singultus

## Abstract

Involuntary diaphragmatic spasms, “hiccups,” commonly resolve spontaneously over a few minutes but can occasionally persist, presenting a challenging perioperative problem. Pregabalin is a frequently utilized gabapentinoid in the treatment of neuropathic pain, but its effectiveness for treating hiccups during the perioperative period has not been well established. We present a patient with a 7‐year history of intractable hiccups resistant to chlorpromazine, baclofen, gabapentin, and cervical epidural injections presenting for orthopedic surgery. Perioperative and postoperative pregabalin administration significantly reduced the frequency and severity of hiccups. This case demonstrates the potential for pregabalin in the management of refractory hiccups.

## 1. Introduction

**FIGURE 1:**
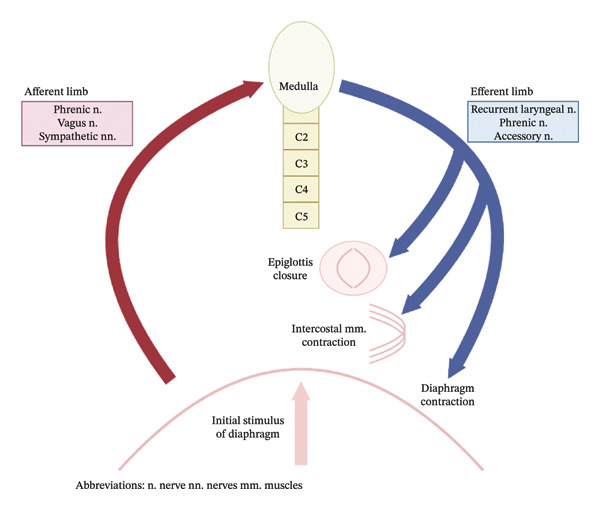
Mechanism of hiccups.

Singultus, also known as hiccups, is a common occurrence that involves an involuntary contraction of the diaphragm and intercostal muscles, and subsequent closure of the glottis. Singultus is classified by duration; acute singultus lasts less than 48 h, persistent singultus lasts over 48 h, and intractable singultus lasts over a month. Severe intractable singultus may stem from neurological, gastrointestinal, cardiovascular, psychiatric, or postoperative causes and can significantly impair sleep, nutrition, social interaction, and quality of life [1]. The primary mechanism of hiccups involves a sudden contraction of the diaphragm, leading to a rapid increase in intrathoracic pressure that forces air against the closed glottis, producing the characteristic “hic” sound (Figure 1). The neural mechanism of hiccups has been elucidated as three afferent neurological pathways: phrenic, vagus, and sympathetic nerves signaling the midbrain [2]. Information from the three neural pathways is processed in the “hiccup center” which includes the medulla, upper spinal cord (C3–C5), reticular formation, and hypothalamus. The “hiccup center” sends three efferent pathways: the recurrent laryngeal nerve to close the glottis, the phrenic nerves to contract the diaphragm, and accessory nerves to contract the intercostal muscles. This continuous diaphragmatic contraction allows for the hiccup loop to occur spontaneously. The neurotransmitter mechanism in singultus is complex and poorly understood, although “hiccup center” control is known to involve serotonin, dopamine, and gamma‐aminobutyric acid (GABA), which serve as popular therapeutic targets. In this report, we describe a complex case of intractable hiccups that spans 7 years is unresponsive to multiple drug regimens and procedures and improves dramatically with pregabalin, highlighting an important perioperative tool for intractable hiccups.

## 2. Consent

Written informed consent for this case report was obtained for the publication of this case report, and the manuscript adheres to applicable EQUATOR guidelines.

## 3. Case Presentation

We present the case of a 61‐year‐old male with a past medical history significant for right acoustic neuroma, hiatal hernia, Type 2 diabetes, prostate cancer, bilateral ulnar mononeuropathies, and persistent intractable hiccups for 7 years of unknown etiology.

Initial pharmacologic management included trials of chlorpromazine, high‐dose baclofen, and gabapentin without sustained benefit. In 2019, the patient underwent three epidural cervical infusions of continuous bupivacaine. He was also prescribed 20 mg of baclofen three times daily, which completely resolved his symptoms temporarily; however in March 2021, he tore his right rotator cuff and fractured several ribs while riding a bicycle. During physical therapy, a neck manipulation triggered a sudden and severe recurrence of his hiccups. Despite continued baclofen therapy, his symptoms persisted. In 2022, further workup for his hiccups revealed a diaphragmatic lesion for which he underwent a video‐assisted thoracoscopic surgery (VATS) to remove a mass. This removal resulted in an improvement of symptoms by 15%–20%. Unfortunately, he continued to experience near constant diaphragmatic spasms with acute exacerbations occurring multiple times per day. Prior to pregabalin initiation, the patient reported hiccups occurring approximately 10–12 times per minute, every hiccup 5‐6 s apart, consistent with near‐continuous diaphragmatic contractions during waking periods.

During the perioperative period for an orthopedic procedure, the patient received a single dose of pregabalin 150 mg orally on the day of the surgery as a part of a standardized multimodal analgesia protocol. Following initiation of pregabalin, the patient experienced a marked reduction in hiccup frequency decreasing from approximately 10–12 episodes per minute to fewer than one episode every several hours. Anesthesia consisted of a single‐syringe total intravenous anesthesia (TIVA) using propofol 10 mg/mL and remifentanil 20 mcg/mL administered at 100 mcg/kg/min adjusted body weight, with a total of 100 mcg fentanyl and placement of a laryngeal mask airway.

On postoperative Day one, the patient received a standardized multimodal pain pack [3], containing celecoxib 100 mg, acetaminophen 625 mg, and pregabalin 50 mg to be taken four times a day for six days. Pregabalin was subsequently maintained postoperatively at 50 mg orally two times daily as maintenance therapy. He experienced side effects such as transient minor drowsiness and mental fogginess, though these improved over time and were tolerable compared to the distress caused by his constant hiccups. At follow‐up 3 years after initiation, the patient continued to report sustained improvement, with hiccup frequency reduced to one to three episodes per hour. He describes this reduction as life‐changing, with significant improvement in sleep, activities of daily living, and overall quality of life.

### 3.1. Clinical Timeline

• 2016: Onset of persistent intractable hiccups (10–12 hiccups per minute)
• 2016–2019: Trials of chlorpromazine, high‐dose baclofen, and gabapentin without sustained improvement
• 2019: Three cervical epidural infusions with transient benefit
• March 2021: Recurrence of intractable hiccups following cervical manipulation
• 2022: VATS for diaphragmatic lesion with partial improvement
• 2022: Orthopedic procedure with initiation of 150 mg pregabalin resulting in marked reduction of hiccup frequency (one hiccup every several hours)
• 2025: Follow‐up at 3 years shows sustained improvement on maintenance pregabalin 50 mg two times per day (one to three hiccups per hour)

## 4. Discussion

Pregabalin is well documented for its use in the treatment of neuropathic pain and is commonly incorporated into multimodal pain regimens in the perioperative setting [3]. However, evidence supporting its use for intractable hiccups in the perioperative period remains limited. Pregabalin is a GABA analog that binds to the Alpha‐2‐delta subunit of voltage‐gated calcium channels, inhibiting calcium influx and thereby reducing excitatory neurotransmitter release in the brain and spinal cord [2]. The proposed mechanism for pregabalin’s efficacy in treating hiccups involves its inhibitory effects on voltage‐gated calcium channels in inspiratory muscles. Given the proposed role of aberrant reflex arc activity involving the phrenic and vagal pathways in hiccups, modulation of central excitatory transmission may attenuate diaphragmatic excitability and suppress the repetitive spasmodic contractions characteristic of hiccups [4].

Gabapentin, which shares a similar mechanism as an Alpha‐2‐delta agonist, is widely considered a first‐line treatment for intractable singultus. However, pregabalin is more potent and absorbs more quickly than gabapentin, offering advantages such as linear absorption within its therapeutic range, more predictable pharmacokinetics, and a smaller dose requirement to achieve therapeutic saturation [5]. These pharmacologic differences may be an important consideration of usage in the perioperative setting particularly due to their potential impact on anesthetic requirements and patient recovery profiles. Some case reports show that a phrenic nerve block can be effective in the resolution of persistent singultus; however, the hiccups often recur once the anesthetic effect subsides [6].

The most common adverse effects of pregabalin involve impaired cognition and coordination, including dizziness, vertigo, and somnolence [7]. While these side effects typically improve with time or dose adjustment, pregabalin carries a risk of misuse due to its GABA‐modulating effects. In adults greater than 65 years old, preoperative gabapentinoid have been linked to delirium and initiation of antipsychotics in the inpatient setting following major surgery [2]. The role of gabapentinoids in the perioperative setting must be carefully individualized to each patient. Some evidence suggests no significant benefit in reducing postoperative pain and highlights increased risks of respiratory depression and abuse [8, 9], while other studies have shown that preemptive gabapentinoid administration can reduce postoperative pain for up to 48 h [10].

A focused literature search was conducted using PubMed through December 2024 using the search terms “pregabalin,” “gabapentin,” “singultus,” and “hiccups.” Eligible publications were limited to English‐language case reports and case series describing pregabalin use for persistent or intractable hiccups. Identified reports are summarized in Table 1. Pregabalin has been successfully utilized in the inpatient, outpatient, and perioperative settings for the treatment of refractory hiccups. Seven of the nine case reports involved patients who had previously trialed other medications such as baclofen, metoclopramide, chlorpromazine, and haloperidol. Reported doses of pregabalin use for hiccup resolution have varied from 25 mg twice daily to 375 mg once daily. In most cases, symptom relief was achieved within 1–3 days, with many patients experiencing complete resolution of hiccups. However, current evidence derives primarily from isolated case reports, limiting assessment of causality and optimal dosing. When conventional therapies fail, a short trial of pregabalin can be considered as an alternative intervention, provided the patient is closely monitored for neurocognitive and respiratory adverse effects. Available evidence remains limited to isolated case reports without standardized outcome measures, precluding conclusions regarding optimal dosing or causality.

**TABLE 1:** Successful cases of intractable singultus treated with pregabalin.

Patient	Patient background	Setting	Description of hiccups	Previous medications attempted	Other methods attempted	Pregabalin management	Outcome	References
1	19 M End‐stage Ewing’s sarcoma, widespread metastatic disease	Inpatient, palliative care	4‐5 h per episode, daily	None, due to exacerbation of sedation in baclofen, chlorpromazine, and haloperidol, refusal of gabapentin due to nonspecific intolerance	None	Day 1: Pregabalin 25 mg BID—no significant improvement Day 2: Pregabalin 50 mg BID—marked improvement	Complete resolution	Groninger 2015 [11]

2	65 M	Outpatient, anesthesia	3 years, continuous	Baclofen 20 mg QD, clonazepam, amitriptyline, haloperidol, chlorpromazine, metoclopramide, Shakuyakukanzoto	C7Th1 cervical epidural block with 5 mL 0.5% lidocaine Right and left phrenic nerve block	Pregabalin 150 mg QD ‐ slight improvement Pregabalin 375 mg QD ‐ marked improvement	Complete resolution	Matsuki 2014 [12]

3	61 M Acute stroke, DM2, HTN, peripheral arteritis	Inpatient, internal medicine	8 days, hiccups attributed to stroke	Haloperidol, baclofen	None	Day 1: Pregabalin 75 mg BID—marked improvement on Day 1, pregabalin stopped at Day 3	Complete resolution	Vandermergel 2006 [13]

4	69 M Arterial HTN, DM2, reflux esophagitis	Outpatient, psychosomatic medicine	20–30 times per minute	Baclofen 40 mg/day metoclopramide, domperidone, tetrazepam, digestive enzymes, and homeopathic products	None	Pregabalin 300 mg QD ‐ slight improvement for 3 months in addition to pantoprazole and baclofen unknown dose and frequency	Bearable 5/10	Jatzko 2006 [14]

5	60 M Arterial HTN, DM2	Outpatient, psychosomatic medicine	15 years, comes and goes every 3‐4 days	Omeprazole, metoclopramide and haloperidol, and homeopathic products	None	100 mg pregabalin QD—marked improvement In addition to omeprazole unknown dose, and frequency	Complete resolution	Jatzko 2006 [15]

6	71 M HTN, HLD, CKD, gastroesophageal disease, cerebral vascular disease, Parkinsonism	Inpatient, neurology	More than 10 years, episode every 2‐3 weeks	None	None	Pregabalin 150 mg QD	Complete resolution	Fong 2020 [16]

7	67 M HTN, HLD, DM2	Inpatient, neurology	3 days, hiccups attributed to stroke	Domperidone, metoclopramide, baclofen	None	Pregabalin 150 mg QD	Complete resolution	Fong 2020 [16]

8	63 M Major depressive disorder	Outpatient clinic, behavioral medicine	4 years, continuous, subsiding during sleep and eating	Haloperidol, risperidone, chlorpromazine	None	Pregabalin 75 mg morning, pregabalin 150 mg evening	Marked improvement, reduced in frequency and duration	Al‐Mahrouqi 2022 [17]

9	67 M	Perioperative, anesthesia	25 days	Baclofen TID Unknown other drugs indicated for hiccups	None	Day 1: Pregabalin 25 mg BID, Day 8: Reduced to 12.5 mg/d	Complete resolution	Nicoletti 2009 [19]

*Note:* DM2, diabetes mellitus Type 2; HTN, hypertension; HLD, hyperlipidemia; QD, daily; BID, twice a day.

Abbreviations: CKD, chronic kidney disease; TID, three times per day.

Perioperative factors including anesthetic choice, opioid administration, and surgical stress response and concomitant analgesic medications may have contributed to the observed reduction in hiccup frequency. In this case, anesthesia consisted of propofol and remifentanil with limited fentanyl exposure. While transient suppression of reflex activity from the anesthesia could theoretically reduce hiccup frequency, these agents have rapid clearance profiles and would not be expected to produce sustained effects beyond the immediate postoperative period. Postoperative management included celecoxib and acetaminophen as a part of a multimodal pain regiment; however, neither medication is known to directly modulate central reflex pathways implicated in singultus. The Alpha‐2 delta–mediated mechanism of pregabalin is not shared by the perioperative or postoperative medications in this case, supporting its role as the primary neuromodulator intervention temporally associated with symptom improvement. Furthermore, the rapid reduction in frequency following pregabalin administration, combined with sustained improvement over 3 years of maintenance therapy makes a purely anesthetic or transient perioperative explanation less likely, though it cannot be excluded.

## 5. Limitations

This report has inherent limitations. As a single case, causality between pregabalin and symptom improvement cannot be established. Symptom frequency was estimated based on patient report and clinical observation rather than singultus severity scales. Regression to the mean, spontaneous fluctuation or placebo effect cannot be excluded. Furthermore, contaminant perioperative medications introduce potential confounding variables. Despite these limitations, the prolonged 7‐year history of near continuous symptoms without sustained remission prior to pregabalin initiation strengthens the temporal association observed. Prospective studies incorporating standardized singultus severity scales and longer follow‐up would be beneficial to determine optimal dosing, safety profile, and generalizability of pregabalin for intractable singultus.

## 6. Conclusion

Intractable singultus presents as a significant perioperative challenge that impacts patient management and comfort. Pregabalin may be useful when other therapies fail to provide adequate relief from persistent hiccups. Despite limited evidence from large‐scale studies, this case report demonstrates that pregabalin offers promising results for hiccup management in various contexts. Addressing persistent hiccups requires comprehensive diagnostic workup and interdisciplinary assessment of potential causes. Clinicians should consider potential adverse effects of pregabalin, particularly in elderly populations due to increased risk of delirium and neurocognitive impairment or those with renal dysfunction. This commonly prescribed perioperative medication has the potential to not only assist in postoperative pain control but also to alleviate some hiccup symptoms, thereby improve perioperative care for our patients.

## Funding

The authors have nothing to report.

## Conflicts of Interest

The authors declare no conflicts of interest.

## Data Availability

The data that support the findings of this study are available from the corresponding author upon reasonable request.
